# Follow that fish: Uncovering the hidden blue economy in coral reef fisheries

**DOI:** 10.1371/journal.pone.0182104

**Published:** 2017-08-03

**Authors:** Shanna Grafeld, Kirsten L. L. Oleson, Lida Teneva, John N. Kittinger

**Affiliations:** 1 Department of Natural Resources and Environmental Management, University of Hawaiʻi at Mānoa, Honolulu, Hawaii, United States of America; 2 Conservation International, Center for Oceans, Honolulu, Hawaii, United States of America; 3 Arizona State University, Center for Biodiversity Outcomes, Julie Ann Wrigley Global Institute of Sustainability, Life Sciences Center, Tempe, Arizona; Department of Agriculture and Water Resources, AUSTRALIA

## Abstract

Despite their importance for human well-being, nearshore fisheries are often data poor, undervalued, and underappreciated in policy and development programs. We assess the value chain for nearshore Hawaiian coral reef fisheries, mapping post-catch distribution and disposition, and quantifying associated monetary, food security, and cultural values. We estimate that the total annual value of the nearshore fishery in Hawaiʻi is $10.3-$16.4 million, composed of non-commercial ($7.2-$12.9 million) and commercial ($2.97 million licensed + $148,500-$445,500 unlicensed) catch. Hawaii’s nearshore fisheries provide >7 million meals annually, with most (>5 million) from the non-commercial sector. Over a third (36%) of meals were planktivores, 26% piscivores, 21% primary consumers, and 18% secondary consumers. Only 62% of licensed commercial catch is accounted for in purchase reports, leaving 38% of landings unreported in sales. Value chains are complex, with major buyers for the commercial fishery including grocery stores (66%), retailers (19%), wholesalers (14%), and restaurants (<1%), who also trade and sell amongst themselves. The bulk of total nearshore catch (72–74%) follows a short value chain, with non-commercial fishers keeping catch for household consumption or community sharing. A small amount (~37,000kg) of reef fish—the equivalent of 1.8% of local catch—is imported annually into Hawaiʻi, 23,000kg of which arrives as passenger luggage on commercial flights from Micronesia. Evidence of exports to the US mainland exists, but is unquantifiable given existing data. Hawaiian nearshore fisheries support fundamental cultural values including subsistence, activity, traditional knowledge, and social cohesion. These small-scale coral reef fisheries provide large-scale benefits to the economy, food security, and cultural practices of Hawaiʻi, underscoring the need for sustainable management. This research highlights the value of information on the value chain for small-scale production systems, making the hidden economy of these fisheries visible and illuminating a range of conservation interventions applicable to Hawaiʻi and beyond.

## Introduction

Small-scale fisheries support food and livelihood security for hundreds of millions of people [1–3], yet suffer from data and information deficiencies that hinder sustainable management. The remote and dispersed nature of these fisheries, along with marginal political power [4], have sidelined the sector from national fisheries development policy [5]. As such, these systems have received far less attention than industrialized fisheries and far fewer resources are devoted to the small-scale fisheries sector. As a result, existing estimates for small-scale fisheries often underestimate their value to local economies. For example, in many Pacific Islands, Zeller found that the contribution of small-scale fisheries to gross domestic product (GDP) was underestimated by factor of five [6] and catches are often substantially greater than previously recognized [7,8].

Sustainable management and fisheries sector development strategies are greatly needed in the US Pacific region, given current challenges to management capacity and the importance of seafood production to local cultures and food security [9–11]. In Hawaiʻi, 75% of nearshore fisheries in the Main Hawaiian Islands (MHI) are in a depleted condition when compared to the protected, unfished Northwestern Hawaiian Islands (NWHI) [12–14] and many commercially harvested species are at 1% of their historical catches. The numerous drivers of these declines include overfishing or harvest below reproductive maturity, the use of unsustainable and/or illegal fishing methods, policies that set minimum sizes below reproductive age, pollution and habitat alteration from coastal development, and several other fisheries-independent factors [13,15,16]. As a result, the economic and sociocultural benefits nearshore fisheries provide to communities have diminished over time.

Managing nearshore reef fisheries in Hawaiʻi for sustainability is challenging due to their diversity and data-poor nature [17]. The current production and catch from these fisheries, as well as their economic value and sociocultural benefits to communities, have not been well quantified until now. While overall it is known that small-scale fisheries in Hawaiʻi generate significant economic benefits [11,18], the details, including the value chains, value-added to GDP, and food provisioning value remain opaque. As a result, it is difficult to develop and implement management programs that are determined by sustainable yields [8,10], or improve returns along the value chain [19]. Further, market value, jobs, and livelihoods are not the only benefits small-scale fisheries deliver, but socio-cultural benefits tend to receive even less attention and are rarely quantified. These include the cultural services and associated use and non-use values [20,21], including sense of place [22,23], heritage and identity [24], social capital [25], and recreational opportunities [26].

In this study, we tracked the flow of fish from catch to consumption and assessed both the monetary and non-monetary benefits of the MHI nearshore reef food fishery. While there is an active fishery harvesting live reef fish for aquarium traders [27], it is outside the scope of this study which exclusively focused on fish caught for consumption. Based on currently available commercial and non-commercial catch estimates, we assess the value chain for nearshore food fisheries (defined as the post-catch distribution and disposition), as well as the monetary value this seafood adds to the economy as it travels through the value chain, the food provisioning potential of the fishery, and the cultural benefits it supports. Our overarching goal is for this assessment to inform the development of strategies to improve monitoring and management and ensure the long-term sustainability of nearshore fisheries in Hawai‘i and beyond.

## Methods

We conducted a value and supply chain assessment, estimating the flows of fish through various distribution chains, together with a valuation to assess the monetary and non-monetary benefits associated with these flows. As such, this study used a mixed methods approach, including value chain tracing, value-added estimations, food provisioning estimates, cultural valuation, field interviews, literature review, and key stakeholder interviews, described in greater detail below.

### Ethics statement

This study was reviewed and approved by the University of Hawaiʻi Human Studies Program Institutional Review Board (study ID CHS22045) prior to beginning. The Landside Operations Manager permitted the research team to conduct surveys at Honolulu International Airport after a lengthy approval process.

### Commercial fishing data

Commercial fishers in Hawai‘i hold fishing licenses, and are required to pay an annual license fee of $50 (2015 cost) and report the catch from every fishing trip by the end of each month, regardless of whether they sold or kept the fish. Annual summaries of these reports are publicly available through the Hawaiʻi Department of Land and Natural Resources (DLNR) Division of Aquatic Resources (DAR) and provide the cumulative weight reported that year with fish organized by Hawaiian common name.

Fish purchasers (i.e., fish dealers), who hold a special marine products license, costing $50 annually, file purchase reports with DAR. While the license is required for any importer, wholesaler, retailer, or restaurant to possess, sell, or offer for sale marine animals restricted by regulations, the reporting of purchases remains voluntary by law. Purchases were analyzed by purchaser type using the four fish dealer categories specified by DAR (Table 1): grocery store, retailer, wholesaler, and restaurant, by island, and by consumer group.

**Table 1:** Description of fish dealer categories according to Hawai‘i DAR.

Fish Dealer Category	Description
**Grocery**	A business that sells primarily food consumption products
**Retail**	A business that sells food and non-food products
**Wholesale**	A business that purchases products with the intention to re-sell them to other dealer categories
**Restaurant**	A business that cooks and sells food.

Purchase reports, which are typically not published, were obtained via a data request submitted to DAR. To protect the confidentiality of fishers and fish dealers, DAR grouped the dataset into five-year (2009–2013) cumulative purchases. This system captures the initial sale of fish to voluntarily reporting commercial purchasers from licensed commercial fishers. Sales from licensed commercial fishers to fish dealers who chose not to participate in optional sales reporting are *de facto* unreported and enter a hidden economy. Unlicensed sales, where unlicensed fishers sell fish to fish dealers, also *de facto* enter the hidden economy as both unreported and illegal catch. Key stakeholders estimated the amount of unlicensed commercial catch entering the market, where fish dealers purchased fish from fishers who did not hold a commercial license, as a percentage of legal sales. Moreover, secondary sales (e.g., from a wholesaler to a grocery store, or from one grocery store to another) are not tracked anywhere. The data obtained from fish dealer purchase reports, therefore, only provides a partial picture of the market transactions along the value chain.

The purchase report dataset used in this study included 57 nearshore fish species organized by Hawaiian common name, island of the sale, dealer category of the purchaser, weight purchased, and total value purchased, as well as the minimum, maximum, and average price paid per kg or landing price. There are several fish with different Hawaiian common names used in the catch and sales datasets. An interview with a DAR employee ensured records were properly matched to fish species. Fish were manually assigned to trophic groups using four categories: planktivores, primary consumers, secondary consumers, and piscivores [28], according to a National Oceanographic and Atmospheric Administration (NOAA) Coral Reef Ecosystem Program delineation (Ivor Williams, pers. comm.). Examples of planktivores include unicornfishes, soldierfishes, and coastal pelagics such as mackerel scad and big-eye scad; primary consumers include surgeonfishes and other herbivores; secondary consumers include triggerfish, goatfish, octopus; piscivore examples are barracuda, jacks, and other predators.

### Market price data

The DAR purchase data include the initial purchase price fish dealers paid directly to licensed commercial fishers (landing price), while market prices, what consumers pay to fish dealers, were compiled from two main sources (S1 Table). NOAA observed average 2009 market prices for the majority of fish in the DLNR dataset [29], while the 2013 values from The Nature Conservancy (TNC) filled in most of the rest [30]. 2013 values were adjusted to 2009 levels based on a 7.8% cumulative inflation rate over the five-year period [31]. After combining these resources, there were still several fish species without published market price data. In these instances, the market price was adjusted. On average, in the grocery sector, market prices were 64% higher than the landing price grocery stores paid fishers; for species which did not have market price data in our data set, we applied a 64% increase based on the species’ landing price (LP_i_) to estimate a likely average market price (MP_i_) (Eq 1). Grocery market prices were used as a standard here for market price estimations because grocery stores were the only dealer type with observed landing prices of all 57 nearshore fish in the DAR commercial sales data. Some fish appear in the non-commercial catch dataset that were not present in the commercial catch dataset and therefore have no purchase price from which to estimate a market value. In these instances, family-level median market prices were used.

$$MP_{i}=LP_{i}+.64LP_{i}$$ (1)

### Non-commercial fishery data

Non-commercial fishing includes subsistence/consumptive, recreational, and cultural fishing and gathering activities that occur in ocean and coastal zones [11,32]. NOAA’s Pacific Islands Fisheries Science Center provides estimates of non-commercial catch as part of the Marine Recreational Information Program (MRIP). We used the annual average weight based on 2004–2011 estimates [33]. There are 136 fish species in this dataset identified by English common name and scientific name.

### Import data: Monitoring at Honolulu International Airport

Following a literature review, which identified a few instances of commercial fish imports [29,34], we determined that there was virtually no information on the non-commercial imports of fish [35]. In-person, semi-structured interviews were conducted to understand the potential small-scale supply of reef fish into Hawaiʻi via air cargo from other Pacific Islands (S1 File). Interviews occurred outside the international arrivals terminal at Honolulu International Airport for a four-week period in October 2015. Flights surveyed arrived from American Samoa, Guam, Marshall Islands, and Samoa. The full sampling protocol is available in supplemental information.

### Key stakeholder interviews

Ten key stakeholders within the fishing industry, academia, and nonprofit sectors with in-depth knowledge about Hawaiʻi’s commercial nearshore fishery across the value chain were interviewed. All of the interviewees were located on Oahu, which is the commercial hub of fishing activity. Several key stakeholders were also actively engaged with fishing communities on Hawaiʻi Island and Maui. Interviews were semi-structured with 5–10 prepared questions for each interviewee based upon their role within the value chain and expected knowledge. Verbal permission to be interviewed was given and no personally identifiable information was collected. Questions addressed the volume of fish in official DLNR sales reports and unreported sales, the relationships between actors in the value chain that are not captured by the current reporting system which only includes the initial purchase of fish from fishers to dealers and includes no secondary trading, and opinions on management. Individual interviews lasted 20–60 minutes, and followed a culturally appropriate (to contemporary society in Hawaii) “talk story” approach, an informal means of interviewing where researchers discuss non-related topics first to establish a rapport with interviewees and then move to an open-ended and fluid discussion of the subject matter [36]. Interviewees provided key insights into the human dimension of the nearshore fisheries value chain in Hawai‘i, validated official DLNR catch and sales data, and provided additional information they felt was not included in official reports. This method elicited estimates of the amount of unreported sales, opinions on current management, and the relationships between stakeholders that influence the structure of the value chain.

### Value chain methods

Value and supply chain analysis evaluates the entire vertical chain of activities from production through processing and distribution to retailing to the consumer [37]. Supply chain analysis focuses on the network of suppliers, retailors, distributors, transporters, and any other actor involved in product supply [38], with an aim to reduce costs, delays, and inefficiencies. Value chain analysis, a particular form of the supply chain approach [39], focuses on the same actors from a market perspective, stressing the importance of value addition in each step, and seeks to understand how value is created and shared among chain participants [40]. Despite the formal differentiation between supply and value chain analysis, the terms are often used interchangeably, and we use value chain for clarity.

To construct the value chain for nearshore fisheries of the MHI, in this study, we:

- Estimated the total nearshore fishery weight and landed value, as well as the scale of unreported commercial sales, with available data on:
  - Commercial fishing catch reports (mandatory filing by all licensed commercial fishers);
  - Fish dealer purchase reports (voluntary reporting by dealers);
  - Fish market prices.
- Semi-quantitatively determined the scale of annual imports and exports of reef fish that may enter or leave the MHI nearshore fisheries value chain:
  - Collecting primary data through key stakeholder interviews at Honolulu International Airport;
  - Searching over 2,000 customer photos and reviews from stores on the US mainland that may be selling nearshore fish on customer review websites such as Yelp.

### Value-added methods

Value-added is defined as the contribution of primary factors of production (i.e., labor and capital) at each step of the value chain, and is measured as the value of output minus the value of all intermediate inputs. Three basic approaches can be used to estimate value-added based upon sector-specific production, income, or expenditures [41]. We used a production-based approach to estimate value-added at the fisher and dealer levels [6,35,42]. This method applies a fixed value-added ratio across the entire fisheries sector [41], although in reality, value-added ratios vary from individual to individual, and are a function of multiple factors, such as fishing skill, business operations, time of year, etc. In the case of fisheries, the value of output is the total sale of catch at ex-vessel price (i.e., the price that fishers receive at port), while the value of intermediate inputs would be costs such as fuel, ice, and vessel maintenance. The difference, which includes income for the fishers and crew and profit for the vessel owner, is the value-added to the economy.

Though simplified, the production approach should return results similar to an income- or expenditure-based approach [41], and while an income/expenditure-based approach would be preferable if detailed financial information were available for the nearshore fishing sector, that level of detail is simply unavailable for most nearshore fisheries, including Hawaii’s. Further, there is precedent in the region for using a production approach, allowing for discussion of Hawaii’s nearshore fishery in a regional context [41]. That said, the assumption underpinning the use of a value-added ratio—i.e., that input costs are a function of output value—is valid only when catch is dependent on fishing effort; this is not always the case in mixed species fisheries, where value can vary independently of input costs. In Hawaiʻi, the market prices for reef fish are similar between species ($11.71/kg sd = $5.60), with the exception of a few species that command a premium price (skewness = 1.77, kurtosis = 6.49). The composition of catch has been relatively stable between years (F = .147, p < .05).

#### Commercial fisher value-added

At the fisher level, value-added for commercial catch (VA_CF_) is the sum of the value-added by species determined by:

$$VA_{CF}=\Sigma_{s}\left(catch_{s}*landing\;price_{s}\right)*value\;added\;ratio_{f}$$ (2)

*Catch*_*s*_ is the annual average weight per species for the 57 commercially caught fish. *Catch* data come from publicly published annual totals for 2009–2013 [43]. *Landing price* is the value from 2009–2013 mean price paid to fishers from data obtained from DAR (S1 Table). For *value-added ratio*, we used a value-added ratio of .60 based upon studies in other Pacific Islands [6,35].

#### Commercial fish dealer value-added

At the dealer level (wholesalers, grocers, retailers, restaurants), value-added (VAC_D_) is the sum of the value-added by species:

$$VA_{CD}=\Sigma_{s}\left(market\;price_{s}-landing\;price_{s}*weight_{s}\right)*value\;added\;ratio_{d}$$ (3)

*Market price* is the observed market prices from previous reports [29,30] and, when unavailable, estimated as described above. *Landing prices* were obtained from the DAR commercial purchase data. The landing price dealers paid for the fish was subtracted from the market value of the fish to avoid double counting the landings value [42]. *Weight* is the annual average weight of fish purchased by dealers as reported in DAR purchase reports [44]. *Value-added ratios* vary by dealer type: wholesalers 0.60, restaurant 0.70, grocery/retail 0.64 based upon national data [45].

These calculations used data on transactions of fish in a flattened, two-step value chain directly from fishers to commercial fish dealers. While there is certainly trade along the value chain, i.e., from fisher to wholesaler/retailer/grocery/restaurant, and between these agents, which would incur markups and associated value-added, there were no available data on trading between dealer types, preventing the inclusion of the value-added from secondary trading. Moreover, several stakeholders concurred that in the MHI reef fishery, most fish dealers sourced directly from fishers.

#### Non-commercial fishing value-added

Hawaii’s non-commercial fishery has been excluded from previous studies estimating the subsistence values of Pacific fisheries due to the claim that Hawaii’s non-commercial fishery is recreational and should therefore be valued as such [6]. This assertion is counter to a broad array of research studies that document the importance of non-commercial nearshore fishing for food provisioning, cultural heritage, and other sociocultural values around consumption, with little evidence of use that is strictly non-consumptive [23,46–51]. As such, we define the non-commercial fishery as a consumptive and subsistence fishery that has a range of values, including recreational value, that defines this diverse fishery.

Potential approaches to valuing non-commercial fishing are farm gate pricing, food provisioning potential, and labor costs. Given the rationale presented above and in line with similar studies in the Pacific, we use “farm gate” pricing [6,41], i.e., the value of subsistence production is estimated as the market price of the fish minus the recurring costs of fishing simplified to a value-added ratio (Eq 4). While the market price is commonly used when estimating “farm gate pricing” [35,41,52,53], this method neglects any value-added by fish dealers. Therefore, we also used the landing price (i.e., the price paid to fishers at the market) to generate a range of the noncommercial fishery’s value. Were we valuing the recreational value of the fishery, we would elect to use labor costs to approximate the opportunity costs of non-commercial fishing. The noncommercial fishery’s value-added (VA_NC_) is estimated as:

$$VA_{NC}=\Sigma_{s}\left(price_{s}*weight_{s}\right)*value\;added\;ratio$$ (4)

Where *price* is either the observed market price from previous reports [29,30] or, when unavailable, estimated as described above, or the landing price obtained from DAR and, when unavailable, estimated as the median landing price by fish family. *Weight* is the annual average catch weight by species [47]. We have applied a *value-added* ratio of non-commercial fishing of 0.90 used in other Pacific Islands [41]. While the larger Hawaiian economy appears substantially different from other Pacific Islands, the fact remains that nearshore fishing substantially contributes to household diets in the archipelago [23,32], especially in rural areas where the commercial value chain is essentially non-functioning and reef fish are absent from local markets and sourced directly through non-commercial sources [34].

### Food provisioning methods

To determine the value of the nearshore catch to food security in the MHI, the average catch was converted from landed weight to edible weight and finally to the equivalent number of meals. Using the method developed by Kittinger et. al.[46] for a non-commercial nearshore fishery on Hawai‘i Island, total weight per species was converted into edible weight and then converted to number of meals using a standardized restaurant portion size of 0.17 grams per serving size. We used the same edible weight conversions as those utilized in the Kittinger et. al. paper [45] and for species that were unavailable in that dataset, contacted the same locally respected fisher and conservationist who provided the estimates in the previous study [45]. This estimation contextualizes the commercial catch in food provision and food security terms, an expression of the value of fisheries beyond its market price.

### Cultural importance methods

Ecosystems provide a range of services to human populations, typically broken down into four broad categories: provisioning, regulating, supporting, and cultural services [54]. Cultural ecosystem services are a useful lens through which to consider human-ecological connections [20,55–58]. However, they are difficult to quantify and incorporate into decision making[20,59], in part because many cultural ecosystem services are incompatible with monetary valuations [60].

As an archipelago in the Pacific, Hawaiʻi has a strong history of nearshore marine resource use that continues today [51,61]. In assessing the cultural values provided by Hawaii’s nearshore fishery, we have drawn on several cultural ecosystem service frameworks [20,59,62,63], particularly one specifically informed by and developed with the inclusion of traditional Hawaiian values from two communities [64], and focus on the subsistence, place/heritage, activity, knowledge, social cohesion, and identity values of Hawaiian nearshore environments. We relied on literature reviews and key stakeholder interviews to identify and assess the provision of cultural ecosystem services.

## Results

### Total production and value of nearshore fisheries

**Fig 1:**
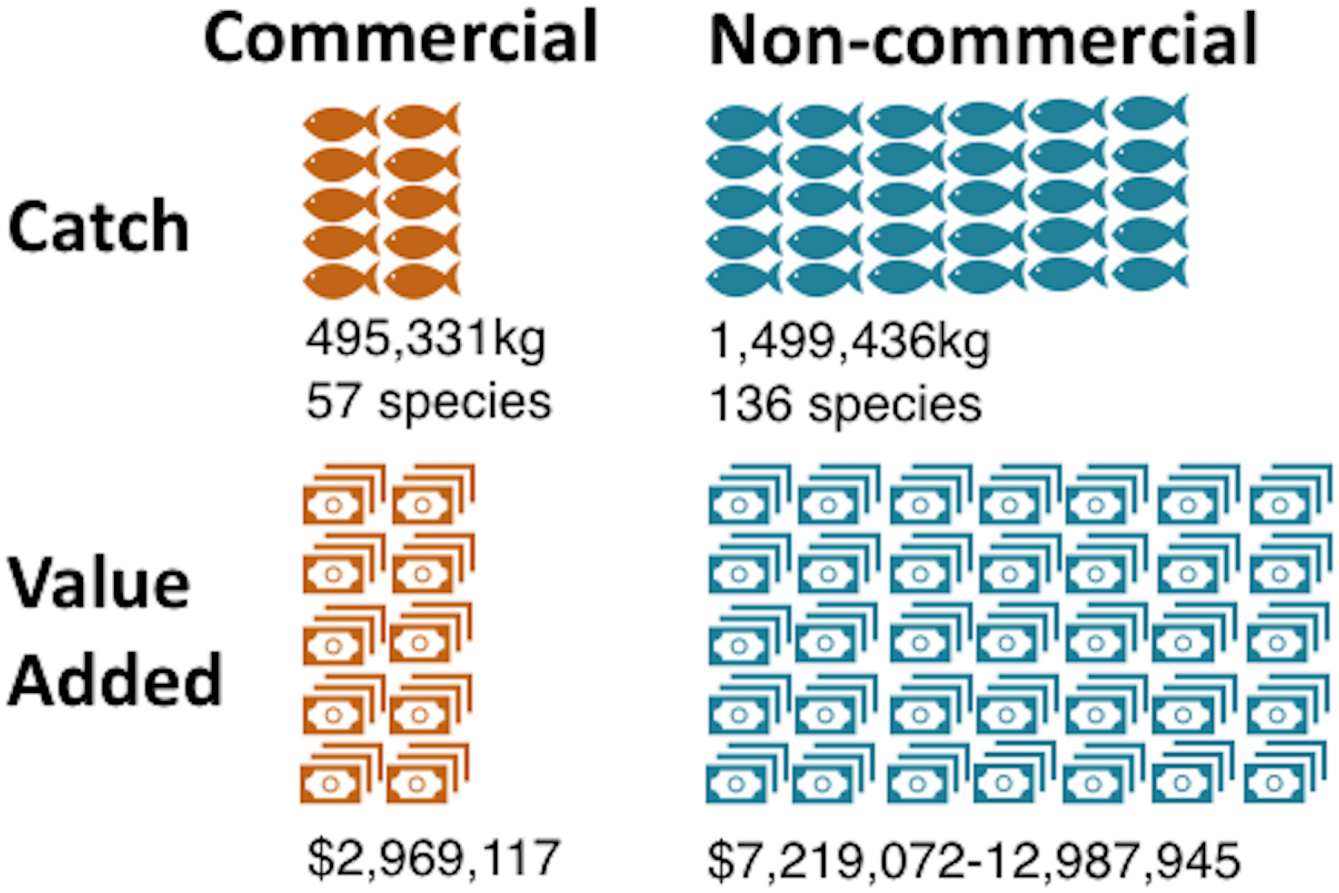
Catch and value-added of commercial and non-commercial nearshore catch in Hawaiʻi.

**Fig 2:**
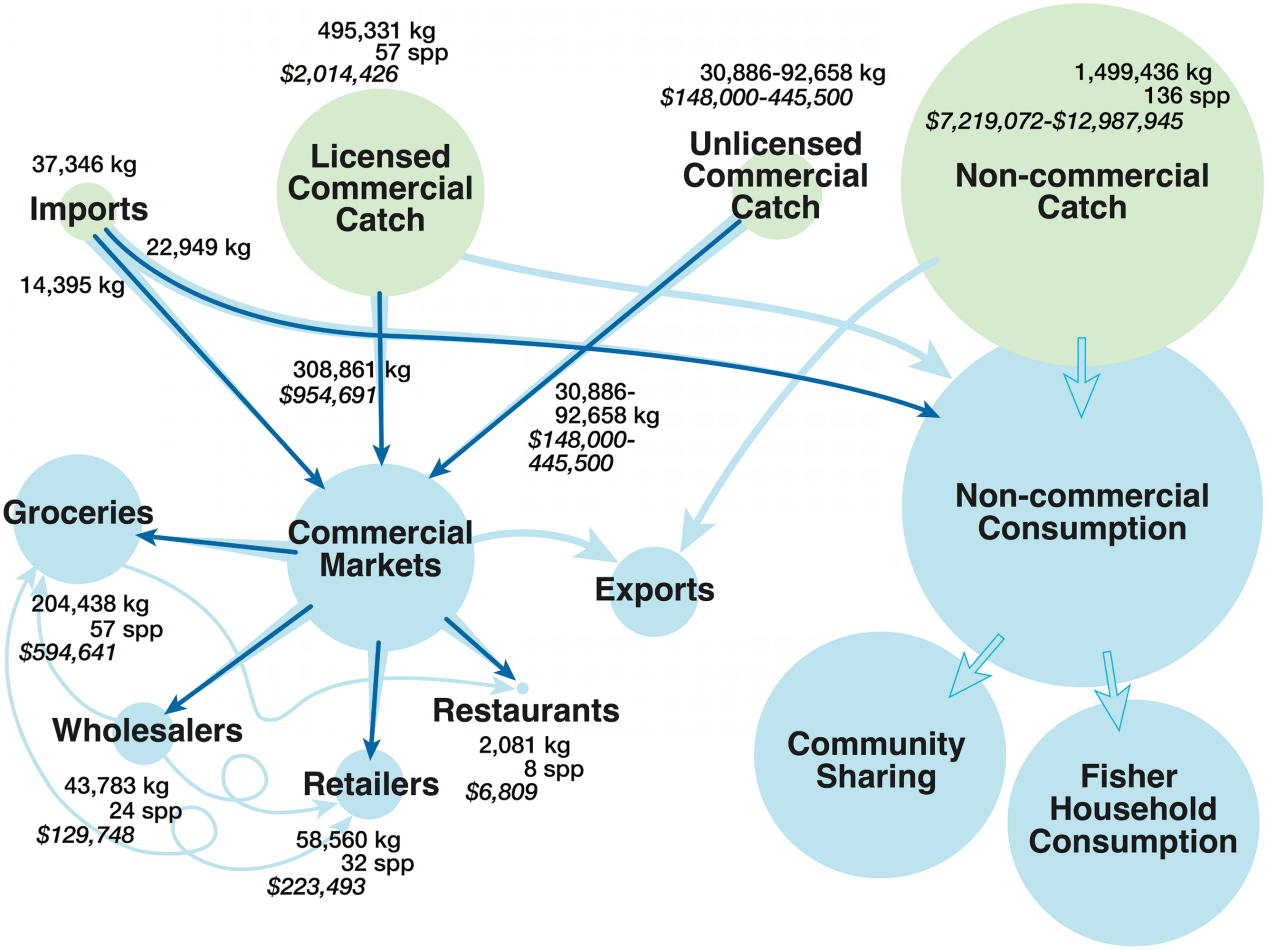
Value chain of nearshore fish in Hawaiʻi. Dark blue arrows represent quantified flows while light blue arrows indicate flows of unknown quantity. Known flows are considered to be underestimates and are nested within larger light blue unknown flows. Production comes from non-commercial and commercial fisheries, with some imports. The non-commercial local fishery largely supplies non-commercial consumption and sharing. Commercial catch derives from both licensed and non-licensed fishers. Only licensed fishers are required to report catch to state officials. Most (at least 62%) of the licensed catch stays in the formal market, and was sold to dealers, who voluntarily report their purchases entering commercial markets directly from fishers. Dealers also trade an undisclosed amount between each other. An unknown amount of nearshore fish is exported from Hawaiʻi. Both the commercial and non-commercial sectors add value to the economy.

Nearly two million kg of nearshore fish were caught annually between 2009–2013, with nearly three times more non-commercial catch (1,499,436, SD = 247,478) than reported commercial catch (495,331kg, SD = 76,304) (Fig 1). Non-commercial catch comprised a greater variety of fish with 136 species versus 57 species for commercial catch. The total annual value-added of the nearshore fishery in Hawaiʻi was approximately $10.3-$16.4 million, with the non-commercial fishery valued between seven (when applying landing price) to nearly 13 million dollars (when applying market price) annually ($7,219,072-$12,987,945). The commercial nearshore fishery added nearly three million dollars ($2,969,117) more to the economy, two million of which was generated at the fisher level, and $954,691 by the four dealer types who reported their purchases directly from fishers (Fig 2): grocery $594,641; retail $223,493; wholesale $129,747; restaurant $6,808. At least 62% of licensed commercial catch remained in the formal economy in commercial markets. The value-added at the dealer level and the proportion of fish in the commercial economy are both conservative estimates derived from the voluntarily reported purchases by fish dealers. The reported, documented flows of fish (indicated by thin, dark arrows in Fig 2) are a subset of larger flows (thicker, lighter arrows in Fig 2) that, while known to exist, have yet to be well documented, thus the value-added cannot be quantified. The value-added estimate also omits value-added from all secondary transactions between dealers (thin, light, squiggly lines in Fig 2). The details of the flows and accompanying values are provided in the sections below.

#### Value chain assessment

**Fig 3:**
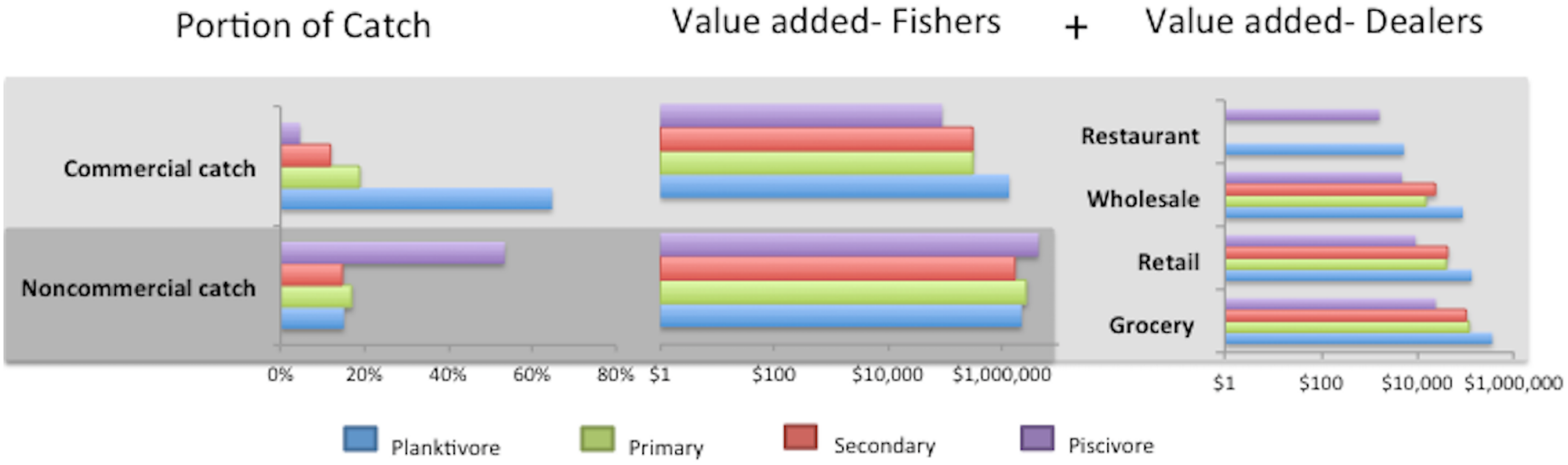
Trophic group breakdowns for commercial and non-commercial catch, by value-added at the fisher level, and additional value-added by commercial fish dealers. Dark gray box demarcates the non-commercial catch and value-added; light gray the commercial catch (value-added derived from fishers and dealers).

Commercial fishers caught 495,169kg (SD = 76,304) annually, while non-commercial fishers caught 1,402,974kg (SD = 247,478). Commercial catch was comprised of planktivores (65%), primary (19%), secondary (12%), and piscivore (4%) consumer groups (Fig 3, panel 1 below). The consumer group breakdown for mean non-commercial catch differed from commercial: planktivore (15%), primary (17%), secondary (15%), piscivore (53%). This catch generated value at the fisher level (Fig 3, panel 2). Some of the commercial catch, 308,275kg (62% of commercial catch), was subsequently reported in DAR purchase reports, while 187,056kg (38%) went unreported. Value was generated by the sale of fish by these dealers (Fig 3, panel 3). Grocery stores purchased both the largest quantity and variety of nearshore fish accounting for 66% of all sales by weight (58,000kg). Grocery stores purchased 57 fish species (58% planktivore, 25% primary, 14% secondary, 3% piscivore by weight). Retailers and wholesalers purchased a similar proportion of total reef fish, 19% and 14%, respectively. Retailers purchased 32 species (66% planktivore, 17% primary, 13% secondary, 4% piscivore by weight). Wholesalers purchased 24 species (69% planktivore, 19% primary, 10% secondary, 3% piscivore). Restaurants reportedly purchased a very small amount, <1% and only purchased eight species (81% planktivore, 0% primary, 0% secondary, 19% piscivore).

The commercial fishery appeared to target primarily coastal pelagic planktivores, as evidenced by planktivores occupying the highest proportion of catch (Fig 3, panel 1); whereas, the non-commercial fishery’s catch was dominated by piscivores in terms of weight. Due to the non-commercial catch exceeding the weight of the commercial catch, the market-equivalent value-added to the economy was greater for the non-commercial fishery compared to the commercial, on average (Fig 3, panel 2). Additional value was added for the portion of commercial catch that was sold to fish dealers (Fig 3, panel 3). Wholesalers, retailers, and grocery stores showed similar preferences in their purchases of amounts of different trophic groups, with planktivorous fishes bringing highest value in all case, followed by secondary consumers, primary consumers, and piscivourous fish.

In addition to purchasing fish directly from fishers, evidence from key stakeholder interviews suggested grocery stores traded seafood amongst themselves and with other dealer categories, particularly wholesalers (Fig 2). Due to lack of data, any additional value-added by secondary trading is outside the scope of our valuations. Conversations with local stakeholders revealed this was important to their business. According to one grocery-sector dealer, there was almost no waste as dealers maintain close business relationships and call one another to sell or purchase fish. There was no space for middlemen in such relationships because the community is tight-knit and the most profit can be generated with little intermediate trading [29].

### Food provisioning capacity

Hawaii’s nearshore fisheries provided over seven million meals annually. The annual commercial fishery catch provided enough fish for 2,363,603 meals while the non-commercial fishery provided an additional 5,332,683 meals. Using species-specific edible weight ratios, when examining “what’s on the plate”, we found that over a third (36%) of meals were planktivores, 25% piscivores, 21% primary consumers, and 18% secondary consumers.

### Imports/Exports

Locally available seafood was supplemented by imports that flow through the commercial and non-commercial chains (Fig 2). Reef species imports and exports were completely missing from the state commercial data, and official import data in the USDA Global Agricultural Trade System [65] were not detailed enough to separate out reef-associated species. Despite the lack of formal data, a 2011 study revealed that almost all commercially imported nearshore fish (estimated at 14,397kg per year) were derived from Asian countries (Taiwan, China, Vietnam, and the Philippines) [34], with the bulk from one dealer who reported importing over 6,803kg of mackerel (*Decapterus macarellus)* per year. Imports of another reef-associated fish, juvenile giant trevally (*Caranx ignobilis*), were also recorded in a past market observation effort [29]. These studies did not observe imports of other commonly eaten coral reef fish families (e.g., parrotfish, wrasse, surgeonfish). We estimated an additional 22,949kg of reef fish is non-commercially imported into Hawaiʻi by means of passenger luggage on passenger flights from Micronesia (Box 1).

**Box 1: Flying fish: Reef fish imports through commercial airline travellers**

A novel effort to track fish imports identified coral reef fish coming into Hawaiʻi from Micronesia, confirming a previous report of air transport of reef fish in the Pacific Region [33]. Intercept surveys at Honolulu airport revealed fish arriving in coolers as luggage on commercial flights from the Marshall Islands (this is an “island hopper” flight with stops in the Federated States of Micronesia: Chuuk, Pohnpei, and the Marshall Islands: Kosrae, Kwajalein, and Majuro before arrival in Honolulu), Guam, and Palau. Additionally, although the Tonga Department of Fisheries has no official records of such exports, there is reportedly transport of reef fish from Tonga to Hawaiʻi [63], however the lack of direct flights between Tonga and Hawaiʻi limited the feasibility of conducting any surveys.

During 15 nights in October 2015, we observed the arrival of 16 flights from the above islands, American Samoa, and Samoa (representing 28% of all flights from these destinations during this time) (S2 Table). 436 coolers were observed arriving off 15 flights. 403 people with coolers were observed and 345 asked for an interview. 98 refused to be interviewed or did not have time to complete an interview. Of the remaining 245, 221 stated the cooler did not contain reef fish (55% of all coolers). Ultimately, 24 individuals confirmed at least one cooler contained reef fish and agreed to be interviewed.

Surveyed individuals brought a mean of 21kg (SD = 22) of nearshore fish with them to Hawaiʻi in 31 coolers. The total mean weight per flight from the Marshall Islands and Federated States of Micronesia was 77kg (SD = 68) and Guam 23kg (SD = 9). Although many individuals arriving from American Samoa or Samoa had coolers, no individuals reported having reef fish with them. All respondents indicated the fish was not for sale, but was for either consumption or sharing. This stream of imports both subsidizes the Hawaiian fish supply, and, as 100% of respondents regarded shipments as culturally important and stated their preference for fish from their island of origin rather than from Hawaiʻi, this imported fish fills an important social and cultural role for immigrant communities.

Applying the mean fish weight reported per flight to the number of flights per year from each destination yields a coarse estimate of the total weight of incoming fish, assuming this one-month sample is representative of a typical non-holiday month. During holiday and graduation seasons (spanning 5 months/year) when large culturally important gatherings are more frequent, respondents estimated that shipments increase by 25%. Based upon the total mean weight, and flight frequency (15/month from Majuro and 25/month from Guam), 1,732kg/month of reef fish was possibly arriving during normal months and 2,165kg/month during holiday seasons. In total, this netted an estimated 22,949kg of reef fish on an annual basis. This is 1.5 times more than previously estimated commercial imports [32]. This estimate represents an innovative attempt to document the flow of reef fish between Pacific Islands through passenger baggage, which is likely an underestimate. The true scale of this hidden economy will not be revealed without coordinated regional effort.

Our research suggests that imports had a relatively small role in the fisheries value chain (Fig 1). Commercial imports (14,395kg) were equivalent to 4.6% of reported commercial sales, or 2.87% of licensed commercial catch. As such, they may have contributed in a small way to the dealers’ economic value. Non-commercial imports (22,949kg) represented less than 2% of estimated locally-sourced non-commercial catch.

Exports of fish from Hawaiʻi to the mainland United States occurred exclusively as air-transport shipments [66]; the vast majority of these shipments consisted of pelagic species (B. Takenaka, United Fishing Agency, pers. comm.). As with imports, reef fish exports did not appear as a separate category in the USDA Global Agricultural Trade System. Based upon this information and confirmation from key fish dealers, it did not appear that large-scale exports of nearshore fish were leaving Hawaiʻi through official channels. Evidence collected in this study suggests, however, that Hawaiʻi reef fishes were shipped to the mainland United States. At least one Oahu-based wholesale company shipped fillets of parrotfish *Scaridae spp*. to consumers on the U.S. mainland. At $37.45/kg, the price for these fillets was over twice the market price in Hawaiian stores, with a minimum order of 8kg. A 2013 advertisement from a Mississippi fish dealer for “Hawaiian uhu” at $59.50/kg, four times the market value in Hawaiʻi, was found via online search, and Hawaiian parrotfish was advertised for sale on the Facebook page of a Chicago area fish dealer. A search of over 2,000 customer photos from Asian/Filipino specialty supermarkets in the Western U.S. on customer review websites, such as Yelp, revealed 10 photos of parrotfish for sale dated from 2007–2014. In every photo with a visible price, the parrotfish was selling for $8.80/kg, less than the average Hawaiʻi market price of $13-16/kg. While the fish was labeled with the United States as its country of origin, upper management did not reply to requests for confirmation.

### Cultural importance

There are many ways in which the MHI reef fishery supports human well being. Subsistence, activity, knowledge, and social cohesion values for fishing in Hawaiʻi were evaluated (Table 2) per the framework adapted from Pascua [64]. The *subsistence value* received by communities encompasses physical sustenance as well as cultural sustenance in the form of the perpetuation of cultural stories, language, traditions, and practices. The *activity value* of the fishery relates to the value people place on participating in fishing. The *knowledge value* of the fishery can be examined through purposeful sharing of fishing knowledge to ensure that traditions are not lost. *Social cohesion* value relates to the value a resource or activity has for fostering trust, a sense of belonging, and cultural norms that ultimately foster the collective, social good [20,25]. Cultural values frequently overlap with each other and may be intertwined with our monetary valuations of commercial and noncommercial fishing. It is therefore possible that some of the value-added estimates already capture some of the cultural values of fish (for example, fishers motivated by physical sustenance, income, and cultural fulfillment from their catch).

**Table 2:** Cultural values, definitions, and evidence in Hawaii.

Value	Definition	Evidence in Hawaii
Subsistence	Physical sustenance from seafood (food)	7.7 million meals annually (this research) Production and sharing from small bay [46] Food motivated non-commercial fishers [50]
	Cultural sustenance from seafood (perpetuation of culture through fishing)	12% of fishers fished to keep traditional practices alive and share fish with the community [50]
Activity	Non-commercial fishing activity	Nearly one-third of residents (out of 1.4 million) fish in Hawaiʻi [67]] Time spent with family/friends, time spent on the ocean, and fishing for food were important motivators of fishing activity [50]
Knowledge	Purposeful sharing of fishing knowledge to ensure that traditions and knowledge are not lost	Community Outreach: Lawaiʻa ʻOhana (Fishing families) Camp Program has been active for six years (2015) and has held 39 camps in 13 communities on 6 islands, welcoming ~2,400 participants 1,076 of whom were children with the goal of sharing traditional fishing knowledge within the community and with future generations [68] Pono (righteous/proper/balanced) practices: NOAA barbless hook outreach efforts [69] Education of 535 children regarding pono fishing (sustainable fishing) and barbless hooks at Nā Kama Kai ocean safety clinics (Bud Antoniles, Nā Kama Kai, pers. comm.)
Social cohesion	Shared norms, mores, and practices related to fishing that promote trust, a sense of belonging, and the collective good	Traditional ahupua’a fisheries management relied upon social cohesion and intergenerational expert knowledge [11,51] Sharing fishing catch within the community is a perpetuation of traditional culture that strengthens social bonds [23,46,48,70]. 78% of recently surveyed fishers report that they always or often shared their catch [50] Fishing knowledge also binds the community, as most fishers (93%) obtained information about fishing and marine issues through their social networks (family and friends) [50]

## Discussion

### Economic value of nearshore fisheries

With a value-added of $10.3-$16.4 million per year, the nearshore fishery (commercial + unlicensed commercial+ non-commercial) comprised up to 8.2% of the total value of all Hawaii’s fisheries. The scale of fisheries operating out of Hawaiʻi has made Honolulu the 6^th^ largest commercial port in the U.S. by catch [45]. Together, the commercial bottomfish fishery valued at nearly $2 million annually [71], the charter recreational fishery, which has been valued at $96.6 million annually (corrected for inflation to 2016 values) [71], the longline fishery of $100 million annual value [72], and the nearshore licensed commercial and non-commercial fishery ($10.3–16.4 million), generate well over $200 million a year.

Notably, the non-commercial nearshore sector generated 2.8 times more fish than the commercial sector, with 2.4–4.4 times more value, and a shorter distribution chain that primarily comprised a sustenance function. This result reflects the lower value-added ratio applied for commercial fishers, and the very small profit margin enjoyed by dealers. It also reflects the different composition of fish targeted by each sector, and the species’ market values. The commercial value-added may also be a significant underestimate, as 38% of licensed commercial catch was not reported in sales, and therefore generated value-added at the fisher level, but not the dealer level, in our estimates. A large portion of the unreported 38% was likely sold, which would generate dealer level value-added, but this was omitted in our estimates. The flow and disposition of unreported commercial seafood sales (38% of the licensed commercial nearshore catch) remains hidden, in part because, at the point of initial sale, commercial dealers are not required to report their purchases or sales to fisheries managers. We also did not have sufficient data to track secondary sales (e.g., wholesaler to retailer), which would likely reveal that the commercial nearshore fishery is even more valuable than our estimate, although because the fishers tend to sell direct to dealers, this is likely not as high as in other places. Further, our licensed commercial estimate ignores an additional $148,500-$445,500 value-added derived from the estimated unlicensed catch that was commercially sold (this estimate assumed the catch composition mimics the licensed). Our estimate of $10.3-$16.4 million annually was solely the direct value-added of the nearshore fishery, however, the indirect and induced effects may be significantly larger as suggested in a recent publication [73].

Placing the nearshore fishery in a regional context, with ~495,000 thousand kg per year worth $3 million in licensed commercial nearshore catch, Hawaiʻi lands more reef fish than any other U.S.-affiliated Pacific island group [10]. However, it ranks 13^th^ overall among 23 Pacific Island Countries and Territories (PICTs) in total weight and total annual value of commercial catch, outpaced by Fiji, Papua New Guinea, Kiribati, Samoa, Tonga, French Polynesia, New Caledonia, and others [41]. When considering non-commercial catch, Hawaiʻi is 7^th^ place by weight, and 8^th^- 12^th^ place by value.

### Subsistence value and vulnerability

We found that nearshore fisheries constitute an important source of sustenance, one which is likely crucially important in rural areas and with economically vulnerable groups. The nearshore fishery annually (2009–2013) provided Hawaiʻi with nearly 7.7 million meals, enough for each of the 1.36 million residents [74] to consume five meals provided by the nearshore environment. Most (70%) of these meals were supplied by the non-commercial fishery sector. These meals highlight the significant yet under-appreciated role that non-commercial fisheries play in food security. The state of Hawai‘i is highly reliant on imports for many food categories, however, in the seafood sector, once local catch is considered, Hawaiʻi imports under 50% of seafood supply consumed in the islands [66]. Such significant contributions to sustenance from seafood are documented in many other parts of the world [7,9,75–77], revealing the need to give greater recognition to the food provisioning role—often a hidden or undervalued one—that fisheries, small-scale agriculture, and other food systems such as forests and hunting play in rural economies [78]. Reef fish is notably absent from markets in rural areas [34], limiting fresh reef fish provisioning to noncommercial fishing and community sharing networks for over 100,000 people living in rural areas. This dependence is particularly strong on the less populated islands (not Oahu) [74], where a larger proportion of people live in rural areas. These communities are also far less affluent, and often a higher proportion are Native Hawaiian.

Historically, some of the highest reef fish catch yields, more than 17mt km^-2^ were back in the 15^th^ century in Hawaii, centuries before European contact [79]. Reef yields have significantly declined following the population boom in the Hawaiian Islands after World War II [79]. Continued nearshore fisheries degradation may have a disproportionate effect on low-income individuals, who may be more sensitive to resource availability as well as price volatility. Volatilities associated with the global and local seafood markets have been observed in other geographies [80]. Residents in Hawai‘i already spend more money purchasing seafood annually than residents of any other U.S. state, not only because seafood is more expensive in Hawai‘i, but also because people in Hawai‘i consume, on average, 80% more seafood than the U.S. national average [66]. Local seafood frequently commands a premium price and is often more expensive than other protein options. If fisheries continue to decline in Hawai‘i, seafood prices may rise, and Hawai‘i residents may become further reliant on imported, processed foods. This shift has occurred in other Pacific Islands, including American Samoa [81]. These protein substitutes are far less healthy and can lead to obesity, diabetes, or other non-communicable diseases [82,83]. Forced by drivers such as demographic change, population increases, influence of markets, new technologies making harvesting more efficient, as well as shifts in institutional governance systems, many Pacific Islands stand poised to experience local resource depletion, with detrimental effects for communities who are especially dependent on local resources [84,85].

### Cultural values and management

Nearshore fisheries support a diverse set of cultural values that can be critical to successfully and sustainably managing the fishery. Successful fisheries management hinges on two understandings: (1) you can directly alter the actions of people, not fish, and knowing what people’s connections are to the fishery is key to designing any interventions [86,87]; and (2) “success” is not a purely ecological condition, but involves humans and their perceptions [88,89]. If benefits are eroded, then people will feel they are losing something of value to them and likely resist management measures.

We have identified several ways in which people genuinely connect to Hawaii’s nearshore fishery. These ecosystem services supported by this fishery include both material subsistence through the provision of local seafood as well as cultural subsistence and the perpetuation of traditional culture through the practice of fishing [64]. Knowledge and social cohesion are both values derived from the fishery, and necessary conditions for effective management [90,91]. Knowledge is actively shared within the fishing community and with the next generation, ensuring the perpetuation of traditional fishing methods and knowledge. Over 1,000 children and their families were educated on sustainable fishing at lawaiʻa ʻohana camps since 2011 [68]. Coordination between NOAA and Nā Kama Kai ocean safety clinics have educated over 500 children on sustainable fishing and marine conservation (Bud Antoniles, pers. comm.). In previous generations, knowledge was primarily gained through watchful study of other fishers [70] with knowledge shared through families, and communities [51]. However, with the shift away from a subsistence lifestyle, is the risk of a loss of the transmission of fishing knowledge [81], increasing the importance of teaching the community about sustainable fishing.

Social cohesion is supported at multiple spatial scales: within families and communities [23], between different areas of the same island [46], between islands in the Hawaiian archipelago [23,46], and between Hawaiʻi and the mainland United States and beyond [23]. Additionally, we have found that fish contribute to social cohesion regionally through exchange between Pacific Islands which may be especially important for the Pacific diaspora. Repondents in our airport surveys said transporting fish was culturally important to them and that sharing fish was an important social norm. Social cohesion may be especially important in ensuring the sustainable use of fishery resources. When communities are involved in fisheries management via community based or co-management strategies, ecological outcomes can be better than areas with “top-down” management strategies such as no take areas that do allow for sustenance harvesting [11].

### Fisheries conservation off the hook: Management and policy implications

**Fig 4:**
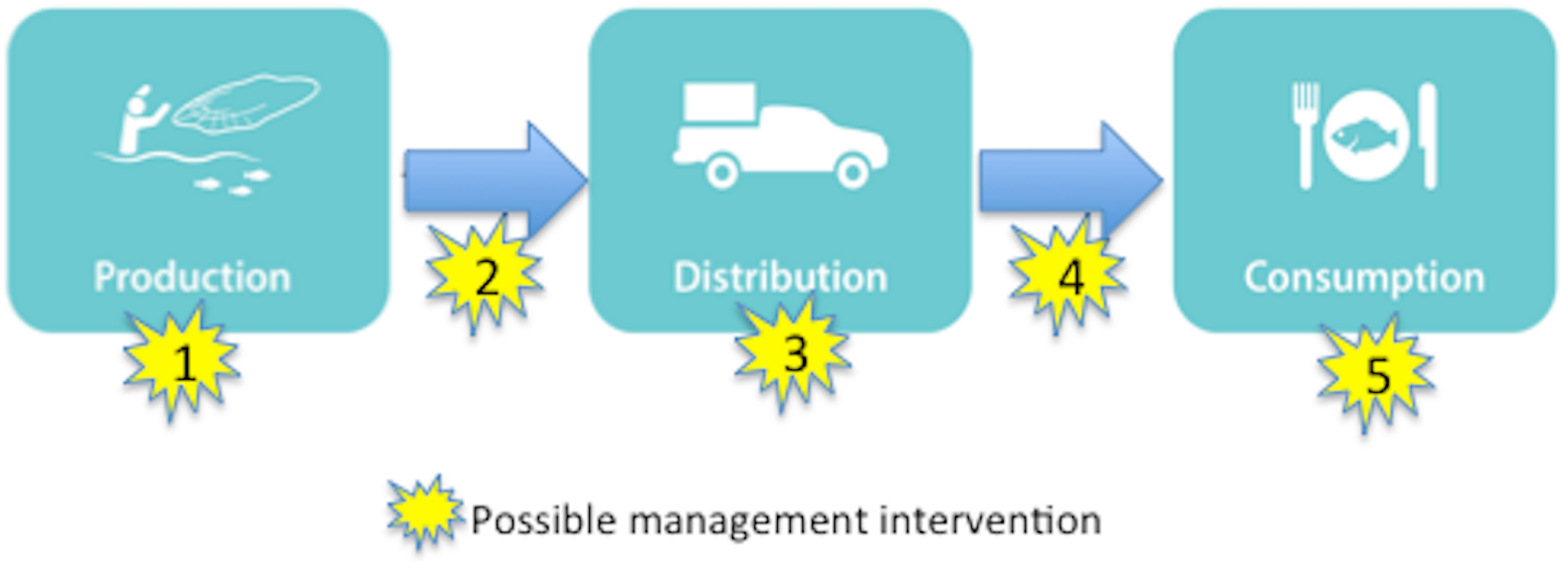
Arrows represent the flow of fish within the supply chain, while stars represent possible intervention points these interventions can occur at the production level (1) e.g. restrictions on fishing activity; distribution level (3) e.g. tracking trading between distributors; consumption level (5) e.g. influencing consumer demand for sustainably caught fish, or at linkages between steps. These linkages include transport to market (2) e.g. improving communication or tracking of fish flows, and the final transport to consumers (4) e.g. mandating reporting for off island shipments.

Given common drivers of fisheries decline such as overfishing, use of unsustainable methods, and harvest of fish below reproductive maturity, fisheries conservation interventions often focus on efforts to protect or enhance the productive capacity of fisheries ecosystems, or control effort by fishers (Star 1 in Fig 4). While these approaches are critical components of any fishery management strategy, our research uncovered a range of conservation interventions that are relevant to the post-catch value and supply chain in Hawaiʻi. Our work adds to a growing body of value/supply chain research [19,80,92,93] suggesting a “conservation off the hook” approach where changes in the market system may enhance social, economic, and ecological outcomes. Careful reflection on the value chain can help reveal key leverage points, where policy interventions could have important, cascading effects [94–96].

Combining the commercial and non-commercial catch revealed that, because the two fisheries target fish types with varying intensity, fishing pressure in Hawaiʻi is spread out across all trophic groups with commercial fishers capturing more low trophic planktivores and non-commercial fishers capturing more high trophic piscivores. While both fisheries are multi-gear and multi-species fisheries operating in the same geographic locations, the targeted species differ. This is likely due to the popularity of planktivores such as bigeye scad in commercial markets, and culturally important bigeye trevally fishing, which puts non-commercial pressure on piscivores. As a result, fishing pressure is found at all trophic levels within Hawaiʻi’s reefs, a common problem all over the world [12,80,87,97]. The constant fishing pressure on all trophic levels may be another reason why ecosystem-based management measures such as marine protected areas (MPAs) could be more effective in reef fisheries where the entire ecosystem requires management [97]. Hawaiʻi currently protects less than 15% of its state waters in MPAs, but the current governor (2017) has committed to effective management of 30% of the nearshore area by 2030 [98].

#### Improved governance

Fisheries management traditionally falls heavily upon production (Star 1, Fig 4), by restricting fishers’ actions without adequately addressing the other actors in the value chain who also have the capacity to influence sustainability. Interventions beyond the fisher level could include such actions as improved monitoring of catch and post-catch distribution (Stars 2 and 3), in order to halt illegal catch (currently estimated at 10–30% the volume of legal commercial catch based upon key stakeholder information) and uncover the extent of unregulated catch. Commercial fishers are bound by rules, in terms of what they catch and where. Illegal fishers may skirt these, potentially causing ecological harm and undermining the social norm of compliance.

In Hawai‘i, mandatory seafood dealer reporting on sales and purchases would allow management agencies to have more accurate assessments of fishing pressure, and allow the market to adjust price points due to more accurately reported supply. Mandatory fish dealer purchase and sale reporting on species of concern in the state’s coral reef fisheries management plan would be particularly useful in aiding state agencies in securing ecological and economic sustainability of the fishery. The data on non-commercial catch could be improved by initiating a licensing scheme, in order to get an estimate of the number of fishers and to be able to design better research on catch. Non-commercial licensing has been proposed in the past, but has met strong resistance from fishers, who perceived it to be a costly step towards the government regulating what they see as their inherent right to fish[99,100].

Additionally, we found that reef fish travel to and from Hawaiʻi in a global economy currently outside of the influence of managers (Star 4 in Fig 4). A small amount (~23,000kg) of reef fish was imported into Hawaiʻi by means of passenger luggage on commercial flights from Micronesia. There was also evidence of shipments of Hawaiian reef fish to the U.S. mainland, although we were unable to validate some of these observations, and data were not available to quantify flows. Better monitoring protocols are needed for nearshore fish imports into Hawaiʻi, and seafood exports out of the archipelago even if they are going to the U.S. mainland. Gaining an understanding of these flows can help inform management, particularly as localized fishing pressure may have a disproportionate effect on some ecologically important species, such as parrotfish [12]. Seafood trade drives exploitation in many locations [80,92,101,102], and the poorly quantified import/export trade in nearshore seafood may be masking notable additions to commercial fishing pressures in Hawaiʻi and its trade partners.

#### Private sector action

Another management action is incentivizing sustainable catch over that which is unsustainable. Sourcing policies that encourage or incentivize fish buyers to prioritize sustainably harvested fish is an action that can promote sustainability within the value chain (Star 3). The value chain for Hawaiian nearshore fisheries (Fig 2) revealed that within the commercial supply chain, there are four major buyer types: grocery stores (66% of reported purchases from fishermen), retailers (19%), wholesalers (14%), and restaurants (<1%); additionally a range of under-reported and undocumented trading and selling occurs. These major buyers are “keystone actors” in the supply chain and may be useful partners in incentivizing sustainable fishing [103]. Rather than trying to change the behavior of 100s or 1000s of fishers, working to affect the purchasing patterns of the major buyers through an improved commitment to sustainable sourcing may be an effective means to improve sustainability [103].

Private sector actors can also improve sustainability by connecting consumers to sustainable sources (Star 4, Fig 4). Community supported fisheries (CSF) which evolved on the concept of community supported agriculture where community members join a co-op and commit to purchasing products on a regular schedule, can shorten the distribution chain, bringing consumers fresh seafood directly from fishers [104]. These arrangements also benefit fishers who have greater market access and the security of pre-arranged purchasers. There is a large CSF operating in California [105] and one on Oahu that has distributed over 2,494kg of local seafood since launching in 2015 [106].

#### Consumer education

A final intervention point focuses on the end of the value chain, educating consumers about sustainable choices (Star 5, Fig 4). Examples include Monterey Bay Aquarium Seafood Watch [107] and certification schemes such as the Marine Stewardship Council that identify sustainable seafood with a logo or seal [108], serving to overcome information asymmetries between consumers and suppliers. Consumer education can also be as simple as signage posted in markets informing consumers about fishing regulations and sustainable practices [92,109].

### The importance of unpacking the market

Recent research explaining the conditions of coral reefs and associated fisheries have focused on the role of “markets”–specifically how accessible reefs are to economic exploitation as an explanatory factor [102]. These analyses often focus on proxies, such as distance to market, or time to get to market. However, our work reveals that the “market” for reef fish is nuanced and complex, and largely driven by non-commercial use, even in a well-developed economy.

Understanding the prevalence of subsistence use is essential, particularly if market pressures are impacting subsistence use [110]. This is critically important, as this value chain provides the most basic benefit to ocean-dependent communities on the planet [3]. The non-commercial fishery captures far more fish and generates more economic value, provides more meals, and supports more cultural values than the commercial fishery. However, non-commercial fishers are unlicensed, and generally less accustomed to top-down management, including information disclosure. Given the distributed nature of the fishery, it is also difficult to design appropriate and targeted management. Although management efforts aimed at non-commercial fishing are often politically difficult due to the cultural importance of the fishery, overcoming these challenges may be necessary to ensure the long-term sustainability of the fishery.

Historical research indicates the productive capacity of Hawaiʻi’s coral reef fisheries has diminished [79], a disheartening status common throughout the world. Rebuilding these fisheries and creating a blueprint for value maximization is an approach that is increasingly popular in the fisheries and aquaculture space [111]. At the same time, managing fisheries for food security, particularly for poor and disadvantaged communities that disproportionately rely on the food provisioning function of these ecosystems, provides a different impetus. Additionally, the cultural values surrounding these fisheries are vast, providing a wellspring of community action and buy-in for localized management [112]

While current fisheries policies and environmental management issues have received some attention from the public and decision makers, policy makers have yet to adequately address data deficiencies or to effectively combine disparate datasets for a comprehensive understanding of nearshore fisheries. Pelagic fisheries in the region are much better understood and studied [113–118], likely because they generate significantly more revenue than nearshore fisheries [35,66]. Pacific pelagic fisheries typically have greater management capacity and are generally producer-driven value chains due to the capital-intensive nature of the fishery, where large-scale industry leaders are central to production [119]. In contrast, nearshore value chains generally resemble buyer-driven value chains where supply networks are decentralized and fish dealers play a much more vital role. Sustainably managing nearshore fisheries is more likely to require managing the entire system in order to maximize the ability to achieve stakeholder goals [88,120]. This, however, necessitates data for the entire social-ecological system, including ecological interactions, human drivers of ecosystem change, and societal benefits.

## Conclusion

Despite the importance of small-scale, nearshore fisheries for human wellbeing in Hawaiʻi and beyond, the sector is notoriously data poor, undervalued, and has less investment in management than other fisheries sectors. In this study, we illuminated the value chain and value-added of Hawaii’s nearshore reef fisheries by combining official catch and sales estimates with key stakeholder information, interview data, import/export observations, cultural ecosystem service estimations, and literature review. We found that value-added of the nearshore fishery is far greater than previous estimates, and suggest that renewed attention to its management is warranted to ensure the longevity of the important economic, social, and cultural benefits of the fishery. The non-commercial sector generates the majority of the nearshore fisheries’ value via a simple and short value chain, limiting potential management responses to focusing on catch, sharing networks, and in-home consumption. A number of value chain interventions in the commercial market could enhance sustainability and economic value-added, e.g., by focusing on connecting producers with distributors. Nearshore fisheries likely contribute to food security of some of Hawaiʻi’s more vulnerable communities and populations. Likewise, the cultural values supported by this fishery are essential to the cultural and social well being of Hawaiʻi residents. Nearshore fisheries are vitally important to millions of people around the world, and bringing hidden blue economies to light improves transparency, allows for a more accurate assessment of value, and ideally will lead to improved management that ensures the sustainable use of fishery resources.

## Supporting information

S1 File Box 1 survey. Survey used to interview individuals transporting reef fish in coolers aboard commercial passenger flights.
(DOCX)

S1 Table Value added data. Economic data used in analysis.
(XLSX)

S2 Table Box 1 data. Data obtained regarding imports of reef fish in coolers.
(XLSX)

## References

[1] Berkes F. Managing small-scale fisheries: alternative directions and methods [Internet]. International Development Research Centre; 2001. https://www.idrc.ca/en/book/managing-small-scale-fisheries-alternative-directions-and-methods

[2] Béné C, Hersoug B, Allison EH. Not by rent alone: analysing the pro-poor functions of small-scale fisheries in developing countries. Dev Policy Rev. 2010;28: 325–358.

[3] Cisneros-Montemayor AM, Pauly D, Weatherdon LV, Ota Y. A Global Estimate of Seafood Consumption by Coastal Indigenous Peoples. PloS One. 2016;11: e0166681 doi: 10.1371/journal.pone.0166681 27918581 10.1371/journal.pone.0166681 PMC5137875

[4] Jacquet J, Pauly D. Funding priorities: big barriers to small-scale fisheries. Conserv Biol. 2008;22: 832–835. doi: 10.1111/j.1523-1739.2008.00978.x 18637910 10.1111/j.1523-1739.2008.00978.x

[5] Chuenpagdee R, Liguori L, Palomares ML, Pauly D. Bottom-up, global estimates of small-scale marine fisheries catches. 2012; https://open.library.ubc.ca/collections/ubccommunityandpartnerspublicati/37052/items/1.0074761

[6] Zeller D, Booth S, Pauly D. Fisheries contributions to the gross domestic product: underestimating small-scale fisheries in the Pacific. Mar Resour Econ. 2006; 355–374.

[7] Barnes-Mauthe M, Oleson KLL, Zafindrasilivonona B. The total economic value of small-scale fisheries with a characterization of post-landing trends: An application in Madagascar with global relevance. Fish Res. 2013;147: 175–185. doi: 10.1016/j.fishres.2013.05.011

[8] Zeller D, Booth S, Davis G, Pauly D. Re-estimation of small-scale fishery catches for US flag-associated island areas in the western Pacific: the last 50 years. Fish Bull. 2007;105: 266–277.

[9] Bell JD, Kronen M, Vunisea A, Nash WJ, Keeble G, Demmke A, et al Planning the use of fish for food security in the Pacific. Mar Policy. 2009;33: 64–76.

[10] Houk P, Rhodes K, Cuetos-Bueno J, Lindfield S, Fread V, McIlwain JL. Commercial coral-reef fisheries across Micronesia: a need for improving management. Coral Reefs. 2012;31: 13–26.

[11] Friedlander AM, Shackeroff JM, Kittinger JN. Customary Marine Resource Knowledge and use in Contemporary Hawai’i. Pac Sci. 2013;67: 441–460.

[12] Friedlander AM, DeMartini EE. Contrasts in density, size, and biomass of reef fishes between the northwestern and the main Hawaiian islands: the effects of fishing down apex predators. Mar Ecol Prog Ser. 2002;230: 253–264.

[13] Williams ID, Walsh WJ, Schroeder RE, Friedlander AM, Richards BL, Stamoulis KA. Assessing the importance of fishing impacts on Hawaiian coral reef fish assemblages along regional-scale human population gradients. Environ Conserv. 2008;35: 261–272.

[14] Nadon MO, Ault JS, Williams ID, Smith SG, DiNardo GT. Length-based assessment of coral reef fish populations in the main and northwestern Hawaiian islands. PloS One. 2015;10: e0133960 doi: 10.1371/journal.pone.0133960 26267473 10.1371/journal.pone.0133960 PMC4534412

[15] Birkeland C. Ratcheting down the coral reefs. BioScience. 2004;54: 1021–1027.

[16] Kronen M, Vunisea A, Magron F, McArdle B. Socio-economic drivers and indicators for artisanal coastal fisheries in Pacific island countries and territories and their use for fisheries management strategies. Mar Policy. 2010;34: 1135–1143.

[17] Kittinger JN, Glazier EW. Special Issue on the Human Dimensions of Small-scale and Traditional Fisheries in the Asia-Pacific Region. University of Hawai’i Press; 2013.

[18] Cesar HS, Van Beukering P. Economic valuation of the coral reefs of Hawai’i. Pac Sci. 2004;58: 231–242.

[19] Jacinto ER, Pomeroy RS. Developing markets for small-scale fisheries: utilizing the value chain approach. Small-Scale Fish Manag Framew Approaches Dev World Cabi Wallingford. 2011; 160–177.

[20] Chan KM, Satterfield T, Goldstein J. Rethinking ecosystem services to better address and navigate cultural values. Ecol Econ. 2012;74: 8–18.

[21] De Groot RS, Wilson MA, Boumans RM. A typology for the classification, description and valuation of ecosystem functions, goods and services. Ecol Econ. 2002;41: 393–408.

[22] Reed M, Courtney P, Urquhart J, Ross N. Beyond fish as commodities: Understanding the socio-cultural role of inshore fisheries in England. Mar Policy. 2013;37: 62–68.

[23] Vaughan MB, Vitousek PM. Mahele: Sustaining Communities through Small-Scale Inshore Fishery Catch and Sharing Networks. Pac Sci. 2013;67: 329–344. doi: 10.2984/67.3.3

[24] Oleson KL, Barnes M, Brander LM, Oliver TA, Van Beek I, Zafindrasilivonona B, et al Cultural bequest values for ecosystem service flows among indigenous fishers: A discrete choice experiment validated with mixed methods. Ecol Econ. 2015;114: 104–116.

[25] Barnes-Mauthe M, Oleson KL, Brander LM, Zafindrasilivonona B, Oliver TA, van Beukering P. Social capital as an ecosystem service: Evidence from a locally managed marine area. Ecosyst Serv. 2015;16: 283–293.

[26] Welcomme RL, Cowx IG, Coates D, Béné C, Funge-Smith S, Halls A, et al Inland capture fisheries. Philos Trans R Soc B Biol Sci. 2010;365: 2881–2896. 10.1098/rstb.2010.0168 PMC2935127 20713391

[27] Tissot BN, Hallacher LE. Effects of aquarium collectors on coral reef fishes in Kona, Hawaii. Conserv Biol. 2003;17: 1759–1768.

[28] Fishbase. The Ecology Table [Internet]. http://www.fishbase.org/manual/FishbaseThe_ECOLOGY_Table.htm

[29] Hospital J, Beavers C. Hawaii retail seafood markets : observations from Honolulu (2007–2011). 2014; 10.7289/V53R0QSM

[30] Mejia M, Warren L, Morishige K, Islesias I. Market Prices of Coral Reef Fish in Hawai`i—Understanding a Valuable Resource. 2014 Aug; Hawaii Conservation Conference.

[31] Coin News LLC. US Inflation Calculator [Internet]. [cited 20 Jul 2016]. http://www.usinflationcalculator.com/inflation/historical-inflation-rates/

[32] Kittinger JN. Participatory Fishing Community Assessments to Support Coral Reef Fisheries Comanagement 1. Pac Sci. 2013;67: 361–381.

[33] Williams I, Ma H. Estimating catch weight of reef fish species using estimation and intercept data from the Hawaii Marine Recreational Fishing Survey. Honol NOAA Pac Isl Fish Sci Cent Adm Rep H-13-04. 2013; 61.

[34] Milne N. Hawaii Coral Reef Dealer Study. Western Pacific Regional Fishery Management Council; 2012.

[35] Gillett R, Lightfoot C. The contribution of fisheries to the economies of Pacific island countries. 2001; https://openaccess.adb.org/handle/11540/2649

[36] Ishida DN, Toomata-Mayer TF, Braginsky NS. Beliefs and attitudes of Samoan women toward early detection of breast cancer and mammography utilization. Cancer. 2001;91: 262–266. 11148591 10.1002/1097-0142(20010101)91:1+<262::aid-cncr16>3.0.co;2-r

[37] Nang’ole E, Mithöfer D, Franzel S. Review of guidelines and manuals for value chain analysis for agricultural and forest products [Internet]. World Agroforestry Centre; 2011. http://dlc.dlib.indiana.edu/dlc/handle/10535/7718

[38] Harland CM. Supply chain management: relationships, chains and networks. Br J Manag. 1996;7: S63–S80.

[39] Hobbs J, Cooney A, Fulton M. Value chains in the Agri-food sector. What Are They. 2000;

[40] van den Berg M, Boomsma M, Cucco I, Cuna L, Janssen N, Moustier P, et al Making Value Chains Work Better for the Poor: A Toolbook for Practitioners of Value Chain Analysis, Version 3 Phnom Penh, Cambodia: Agricultural Development International; 2009.

[41] Gillett R. Fisheries in the economies of the Pacific island countries and territories [Internet]. 2nd ed Asian Development Bank; 2016 https://www.spc.int/DigitalLibrary/Doc/FAME/Manuals/Gillett_16_Benefish.pdf

[42] Leung P, Loke M. The contribution of agriculture to Hawai “i”s economy: 2005. Econ Issues. 2008;13. http://www.ctahr.hawaii.edu/oc/freepubs/pdf/EI-13.pdf

[43] State of Hawaii Department of Land and Natural Resources. Commercial Fishing. In: Division of Aquatic Resources [Internet]. [cited 5 Jan 2016]. http://dlnr.hawaii.gov/dar/fishing/commercial-fishing/

[44] State of Hawaii Department of Land and Natural Resources. Hawaii Commercial Marine License Dealer Reports (2004–2008, 2009–2013). 2014.

[45] National Marine Fisheries Service. Summary of 2011 Value-added, margins, and consumer expenditures for commercial marine fishery products in the United States [Internet]. National Oceanic and Atmospheric Administration Washington, DC; 2011. https://www.st.nmfs.noaa.gov/Assets/commercial/fus/fus11/09_econ2011.pdf

[46] Kittinger JN, Teneva LT, Koike H, Stamoulis KA, Kittinger DS, Oleson KL, et al From reef to table: Social and ecological factors affecting coral reef fisheries, artisanal seafood supply chains, and seafood security. PloS One. 2015;10: e0123856 doi: 10.1371/journal.pone.0123856 26244910 10.1371/journal.pone.0123856 PMC4526684

[47] McCoy K. Estimating Nearshore Fisheries Catch for the Main Hawaiian Islands. University of Hawaii at Manoa. 2015.

[48] Severance C. Customary exchange maintains cultural continuity. Pac Isl Fish News Summer. 2010; 1–2.

[49] Glazier E, Carothers C, Milne N, Iwamoto M. Seafood and Society on O’ahu in the Main Hawaiian Islands 1. Pac Sci. 2013;67: 345–359.

[50] Madge L, Hospital J, Barbier EB. Attitudes and Preferences of Hawaii Non-commercial Fishers: Report from the 2015 Hawaii Saltwater Angler Survey. U.S. Dep. Commer.; 2016 p. 36 p. + Appendices. Report No.: NOAA Tech. Memo., NOAA-TM-NMFS-PIFSC-58.

[51] Jokiel PL, Rodgers KS, Walsh WJ, Polhemus DA, Wilhelm TA. Marine resource management in the Hawaiian Archipelago: the traditional Hawaiian system in relation to the Western approach. J Mar Biol. 2011;2011 http://www.hindawi.com/journals/jmb/2011/151682/abs/

[52] Fafchamps M, Hill RV. Selling at the Farmgate or Traveling to Market. Am J Agric Econ. 2005;87: 717–734.

[53] Bain D. A guide to estimating the value of household non-market production in Pacific island developing countries. South Pacific Commission; 1996.

[54] Millennium Ecosystem Assessment. Ecosystems and human well-being. Wash DC. 2005; https://www.pik-potsdam.de/members/cramer/teaching/06/mea_jrock.pdf

[55] Darvill R, Lindo Z. Quantifying and mapping ecosystem service use across stakeholder groups: Implications for conservation with priorities for cultural values. Ecosyst Serv. 2015;13: 153–161.

[56] Liu J, Opdam P. Valuing ecosystem services in community-based landscape planning: introducing a wellbeing-based approach. Landsc Ecol. 2014;29: 1347–1360.

[57] Liu J, Dietz T, Carpenter SR, Alberti M, Folke C, Moran E, et al Complexity of coupled human and natural systems. science. 2007;317: 1513–1516. doi: 10.1126/science.1144004 17872436 10.1126/science.1144004

[58] Plieninger T, Bieling C, Fagerholm N, Byg A, Hartel T, Hurley P, et al The role of cultural ecosystem services in landscape management and planning. Curr Opin Environ Sustain. 2015;14: 28–33.

[59] Daily GC, Polasky S, Goldstein J, Kareiva PM, Mooney HA, Pejchar L, et al Ecosystem services in decision making: time to deliver. Front Ecol Environ. 2009;7: 21–28.

[60] Bunse L, Rendon O, Luque S. What can deliberative approaches bring to the monetary valuation of ecosystem services? A literature review. Ecosyst Serv. 2015;14: 88–97.

[61] Poepoe KK, Bartram PK, Friedlander AM. The use of traditional Hawaiian knowledge in the contemporary management of marine resources. Fish Cent Res Rep. 2003;11: 328–339.

[62] Daniel TC, Muhar A, Arnberger A, Aznar O, Boyd JW, Chan KM, et al Contributions of cultural services to the ecosystem services agenda. Proc Natl Acad Sci. 2012;109: 8812–8819. doi: 10.1073/pnas.1114773109 22615401 10.1073/pnas.1114773109 PMC3384142

[63] Hernández-Morcillo M, Plieninger T, Bieling C. An empirical review of cultural ecosystem service indicators. Ecol Indic. 2013;29: 434–444.

[64] Pascua P ʻala. I ola ka ʻāina, i ola nō kākou: place-based and indigenous perspectives on cultural ecosystem services in Hawaiʻi. University of Hawaii at Manoa. 2015.

[65] United States Department of Agriculture. Global Agricultural Trade System [Internet]. http://apps.fas.usda.gov/GATS/default.aspx

[66] Geslani C, Loke M, Takenaka B, Leung P. Hawaii’s seafood consumption and its supply sources. Jt Inst Mar Atmospheric Res SOEST Publ. 2012;12. http://www2.hawaii.edu/~geslani/files/Leung_HIseafood.final.pdf

[67] OmniTrack Group. 2011 Seafood Security Study. 2011.

[68] Conservation International Hawaii. Lawaiʻa ʻOhana Camp Program Developing the next generation of pono fishers in Hawaiʻi Fact Sheet. 2016.

[69] PIFSC. Barbless Circle Hook News [Internet]. NOAA Fisheries; 2016. https://www.pifsc.noaa.gov/barbless_circle_hook/

[70] Kohala Center. Health Impact Assessment of the proposed Mo‘omomi Community-Based Subsistence Fishing Area [Internet]. http://www.pewtrusts.org/~/media/assets/external-sites/health-impact-project/kohala-2016-moomomi-fishing-area-final-report.pdf?la=en

[71] Western Pacific Regional Fishery Management Council. Bottomfish Fisheries in the Hawaii Archipelago [Internet]. 2013. http://www.wpcouncil.org/wp-content/uploads/2013/04/2010-Bottomfish-Fisheries-in-the-Hawaii-Archipelago-Fact-Sheet-FINAL-5-06-10.pdf

[72] National Marine Fisheries Service Lowther. Fisheries of the United States: 2014 [Internet]. Government Printing Office; 2016. https://www.st.nmfs.noaa.gov/Assets/commercial/fus/fus13/FUS2013.pdf

[73] Lovell SJ, Steinback SR, Hilger JR. The economic contribution of marine angler expenditures in the United States, 2011. 2013.

[74] Hawaii Department of Business, Economic Development & Tourism. Urban and Rural Areas in the State of Hawaii, by County: 2010 [Internet]. Hawaii State Data Center; 2013 Sep p. 11. Report No.: HSDC 2013–2. http://files.hawaii.gov/dbedt/census/Census_2010/Other/2010urban_rural_report.pdf

[75] Béné C, Friend RM. Poverty in small-scale fisheries: old issue, new analysis. Prog Dev Stud. 2011;11: 119–144.

[76] Loring PA, Gerlach SC, Harrison HL. Seafood as local food: Food security and locally caught seafood on Alaska’s Kenai Peninsula. J Agric Food Syst Community Dev. 2016;3: 13–30.

[77] Smith MD, Roheim CA, Crowder LB, Halpern BS, Turnipseed M, Anderson JL, et al Sustainability and Global Seafood. Science. 2010;327: 784–786. doi: 10.1126/science.1185345 20150469 10.1126/science.1185345

[78] Godfray HCJ, Beddington JR, Crute IR, Haddad L, Lawrence D, Muir JF, et al Food security: the challenge of feeding 9 billion people. science. 2010;327: 812–818. doi: 10.1126/science.1185383 20110467 10.1126/science.1185383

[79] McClenachan L, Kittinger JN. Multicentury trends and the sustainability of coral reef fisheries in Hawai ‘i and Florida. Fish Fish. 2013;14: 239–255.

[80] Thyresson M, Crona B, Nyström M, de la Torre-Castro M, Jiddawi N. Tracing value chains to understand effects of trade on coral reef fish in Zanzibar, Tanzania. Mar Policy. 2013;38: 246–256.

[81] Levine A, Allen S. American Samoa as a fishing community [Internet]. 2009. https://pifsc-www.irc.noaa.gov/library/pubs/tech/NOAA_Tech_Memo_PIFSC_19.pdf

[82] Rausser G, Hamilton S, Kovach M, Stifter R. Unintended consequences: The spillover effects of common property regulations. Mar Policy. 2009;33: 24–39.

[83] Wellman KF. The US retail demand for fish products: an application of the almost ideal demand system. Appl Econ. 1992;24: 445–457.

[84] Cinner JE, Daw T, McClanahan TR. Socioeconomic factors that affect artisanal fishers’ readiness to exit a declining fishery. Conserv Biol. 2009;23: 124–130. doi: 10.1111/j.1523-1739.2008.01041.x 18778267 10.1111/j.1523-1739.2008.01041.x

[85] Mora C, Myers RA, Coll M, Libralato S, Pitcher TJ, Sumaila RU, et al Management effectiveness of the world’s marine fisheries. PLoS Biol. 2009;7: e1000131 doi: 10.1371/journal.pbio.1000131 19547743 10.1371/journal.pbio.1000131 PMC2690453

[86] Cinner JE, Pollnac RB. Poverty, perceptions and planning: why socioeconomics matter in the management of Mexican reefs. Ocean Coast Manag. 2004;47: 479–493.

[87] Crona B, Bodin Ö, others. Power asymmetries in small-scale fisheries: a barrier to governance transformability. Ecol Soc. 2010;15: 32.

[88] Weijerman M, Grace-McCaskey C, Grafeld SL, Kotowicz DM, Oleson KLL, van Putten IE. Towards an ecosystem-based approach of Guam’s coral reefs: The human dimension. Mar Policy. 2016;63: 8–17. doi: 10.1016/j.marpol.2015.09.028

[89] Cinner JE, McClanahan TR, MacNeil MA, Graham NA, Daw TM, Mukminin A, et al Comanagement of coral reef social-ecological systems. Proc Natl Acad Sci. 2012;109: 5219–5222. doi: 10.1073/pnas.1121215109 22431631 10.1073/pnas.1121215109 PMC3325732

[90] Gutiérrez NL, Hilborn R, Defeo O. Leadership, social capital and incentives promote successful fisheries. Nature. 2011;470: 386–389. doi: 10.1038/nature09689 21209616 10.1038/nature09689

[91] Ostrom E. Governing the commons [Internet]. Cambridge university press; 2015 https://books.google.com/books?hl=en&lr=&id=daKNCgAAQBAJ&oi=fnd&pg=PR11&dq=Elinor+Ostrom&ots=Ycqjmwf46y&sig=ZyekN1vN-onibuHBufyd3iZ9Grw

[92] Brewer TD. Coral reef fish value chains in Solomon Islands: Market opportunities and market effects on fish stocks. ARC Cent Excell Coral Reef Stud Rep Solomon Isl Minist Fish Mar Resour Secr Pac Community. 2011;46. http://www.spc.int/DigitalLibrary/Doc/FAME/Reports/Brewer_11_FishValue_SolomonIs.pdf

[93] Brown E., Perez M.L., Garces L.R., Ragaza R.J., Bassig R.A., Zaragoza E.C.. Value Chain Analysis for Sea Cucumber in the Philippines. The WorldFish Center; 2010.

[94] Schmitz H. Value chain analysis for policy-makers and practitioners [Internet]. International Labour Organization; 2005 https://books.google.com/books?hl=en&lr=&id=oclHmtDrwLoC&oi=fnd&pg=PR3&dq=Value+chain+analysis+for+policy-makers+and+practitioners.&ots=NjGTivPX40&sig=nAqqEH7qxNZ4uVPFMzyYlcQnnr0

[95] Nguyen NC, Bosch OJ. A systems thinking approach to identify leverage points for sustainability: a case study in the Cat Ba Biosphere Reserve, Vietnam. Syst Res Behav Sci. 2013;30: 104–115.

[96] Meadows D. Leverage points. Places Interv Syst. 1999; http://drbalcom.pbworks.com/w/file/fetch/35173014/Leverage_Points.pdf

[97] Soler GA, Edgar GJ, Thomson RJ, Kininmonth S, Campbell SJ, Dawson TP, et al Reef Fishes at All Trophic Levels Respond Positively to Effective Marine Protected Areas. PLOS ONE. 2015;10: e0140270 doi: 10.1371/journal.pone.0140270 26461104 10.1371/journal.pone.0140270 PMC4603671

[98] State of Hawaii. Natural Resource Management, Target: Increase Marine Management in Hawaii [Internet]. 2017. https://dashboard.hawaii.gov/en/stat/goals/5xhf-begg/4s33-f5iv/ydtj-mhwg/view

[99] Kevin Chang, Eric Co, Joshua DeMello, Frank Farm, Phil Fernandez, Aarin Gross, et al. Feasibility of a Non-Commercial marine Fishing Registry, Permit, or License System in Hawaii [Internet]. State of Hawaii DLNR; 2016. https://dlnr.hawaii.gov/dar/files/2016/12/NCMF_final_report.pdf

[100] Max Dible. Study looks at feasibility of implementing non-commercial fishing licensing system. West Hawaii Today. 3 Dec 2016. http://hawaiitribune-herald.com/news/local-news/study-looks-feasibility-implementing-non-commercial-fishing-licensing-system

[101] Sadovy YJ, Donaldson TJ, Graham TR, McGilvray F, Muldoon GJ, Phillips MJ, et al While stocks last: The live reef food fish trade. 2003; https://think-asia.org/handle/11540/2432

[102] Cinner JE, Graham NA, Huchery C, Macneil MA. Global effects of local human population density and distance to markets on the condition of coral reef fisheries. Conserv Biol. 2013;27: 453–458. doi: 10.1111/j.1523-1739.2012.01933.x 23025334 10.1111/j.1523-1739.2012.01933.x

[103] Österblom H, Jouffray J-B, Folke C, Crona B, Troell M, Merrie A, et al Transnational Corporations as “Keystone Actors” in Marine Ecosystems. PLOS ONE. 2015;10: e0127533 doi: 10.1371/journal.pone.0127533 26017777 10.1371/journal.pone.0127533 PMC4446349

[104] Brinson A, Lee M-Y, Rountree B. Direct marketing strategies: the rise of community supported fishery programs. Mar Policy. 2011;35: 542–548.

[105] Local Bounty Inc. Real Good Fish [Internet]. http://www.realgoodfish.com

[106] Local I’a Hawaii. Local I’a Hawaii [Internet]. http://localiahawaii.com

[107] Seafood Watch. Monterey Bay Aquarium Foundation; 2017. https://www.seafoodwatch.org/seafood-recommendations

[108] Marine Stewardship Council. Certified Sustainable Seafood [Internet]. 2017. https://www.msc.org

[109] Cedric B. Tanzanian Nile Perch Value Chain Baseline Study [Internet]. Rongead International Trade & Sustainable Development; 2013 p. 17. http://www.rongead.org/IMG/pdf/Rongead_Rapport_Final.pdf

[110] Cinner J, Huchery C, MacNeil MA, Graham NA, McClanahan TR, Maina J, et al Bright spots among the world’s coral reefs. Nature. 2016;535: 416–419. doi: 10.1038/nature18607 27309809 10.1038/nature18607

[111] California Environmental Associates. Ocean Prosperity Roadmap: Fisheries and Beyond [Internet]. 2015. http://www.oceanprosperityroadmap.org/wp-content/uploads/2015/05/Synthesis-Report-6.14.15.pdf.

[112] Ayers A, Kittinger J, Imperial M, Vaughan M. Making the transition to co-management governance arrangements in Hawai‘i: a framework for understanding transaction and transformation costs. Int J Commons. 2017;11 doi: 10.18352/ijc.709

[113] Miller ML, Kaneko J, Bartram P, Marks J, Brewer DD. Cultural consensus analysis and environmental anthropology: Yellowfin tuna fishery management in Hawaii. Cross-Cult Res. 2004;38: 289–314.

[114] Hampton J, Langley A, Kleiber P. Stock assessment of bigeye tuna in the western and central Pacific Ocean, including an analysis of management options. WCPFC SC2 SA WP-2 Manila Philipp. 2006;7: 18.

[115] Hannesson R, Kennedy J. Rent-maximization versus competition in the western and central pacific tuna fishery. J Nat Resour Policy Res. 2008;1: 49–65.

[116] Fernandes da Costa PM, Hu W, Pan M. Consumer demand for ahi poke (raw tuna salad) in Hawaii. 2010 Annual Meeting, February 6–9, 2010, Orlando, Florida. Southern Agricultural Economics Association; 2009. http://ideas.repec.org/p/ags/saea10/56346.html

[117] Barnes-Mauthe M, Arita S, Allen SD, Gray SA, Leung P. The influence of ethnic diversity on social network structure in a common-pool resource system: implications for collaborative management. Ecol Soc. 2013;18: 23–36.

[118] Richmond L, Kotowicz D, Hospital J. Monitoring socioeconomic impacts of Hawai “i”s 2010 bigeye tuna closure: Complexities of local management in a global fishery. Ocean Coast Manag. 2015;106: 87–96.

[119] Hempel E. Value chain analysis in the fisheries sector in Africa. Note Study Carried Collab INFOSA Funded Trade Work Group Partnersh Afr Fish AUNEPAD Programme. 2010; http://www.fao.org/fileadmin/user_upload/fisheries/docs/Value_Chain_Analysis_Report_FINAL_hempel.doc

[120] Hilborn R, Ovando D. Reflections on the success of traditional fisheries management. ICES J Mar Sci J Cons. 2014;71: 1040–1046.

