# Supplementary material for: Follow that fish: Uncovering the hidden blue economy in coral reef fisheries
Source: PLoS One. 2017 Aug 3;12(8):e0182104. doi: 10.1371/journal.pone.0182104 (PMC5542444; doi:10.1371/journal.pone.0182104)
Supplement: S1 File — Survey used to interview individuals transporting reef fish in coolers aboard commercial passenger flights. (DOCX) [file pone.0182104.s001.docx]

Box 1: Flying Fish Survey Methods

Survey methods were reviewed by the University of Hawaiʻi Human Studies Institutional Review Board prior to beginning and found to be exempt. Verbal permission to be interviewed was given by respondents, but no personally identifiable information was collected. Sampling captured 15 flights out of 53 arrivals from these destinations during the four-week period. Sampling days were determined by flight schedules. For routes with multiple arrivals per week (e.g., Marshall Islands flights arrive on Mondays, Wednesdays and Fridays), each week-day was surveyed at least once. Working in a team of 2-3 researchers each time a flight was met, one researcher counted the number of coolers arriving off a flight while the remaining researcher(s) approached potential respondents. Respondents were first asked if they had nearshore fish in their cooler. If they said yes, and agreed to be interviewed, they were asked a standard list of questions to gauge the approximate quantity of fish, where the fish was caught, the purpose of bringing the fish to Hawaiʻi, and the cultural importance of this action. Questions regarding fish sales were also prepared, but were not used as no respondents stated an intention to sell their fish. Respondents were then shown a laminated card depicting “Common Food Fishes of Micronesia” [1] and were asked to circle the type of fish they were transporting using a dry erase marker. In interviews on the first day of sampling, the laminated card displayed Hawaiian reef fish, which one respondent noted was inappropriate given the origin of the fish, prompting the change to the CNMI card. Fish were coded to corresponded with a number 1-50. The researcher conducting the interview noted down the number associated with each fish selected by the interviewee. Each interview lasted less than five minutes and no identifying information was collected about respondents. Data were organized and analyzed using Excel. A sample interview sheet follows.

Date: Arrival From:

Verbal permission to be surveyed: Y/N (If no, circle N and do not continue survey)

Reef fish present in cooler? Y/N (If no, circle N and do not continue survey)

Where was fish caught?

Where do you live?:

IF “HAWAII”: Is it important for you culturally to eat fish from (CNMI/MI/AS)?

Did you bring this fish to share with your family/friends?

Is this fish for a special occasion like a wedding or birthday party?

How many trips a year do you bring reef fish to Hawaii?

Do you know the weight of fish you brought on this trip?

Do you usually bring about this much? Less? More?

Are there any differences between the fish from (CNMI/MI/AS) and Hawaii?

Do you prefer to eat fish from (CNMI/MI/AS) or Hawaii? Why?

Is it important for you culturally to bring fish to share in Hawaii?

If you didn’t bring this fish with you, how would that affect you/your family?

*Ask respondent to circle the fish they have brought with them, and record the fish circled below.

**Sales**

How many trips a year do you bring reef fish to sell in Hawaii?

Do you know how many lbs of fish you brought on this trip?

Do you usually bring about this much? Less? More?

Did you bring enough fish to pay for your trip to Hawaii? Less? More?

Which times of year, if any, are especially important for you to bring fish to sell? Why?

What % of fish do you sell directly to consumers? % to fish traders? % to store?

Which species are the most valuable for you to sell in Hawaii?

## Citations

1. Commonwealth of the Northern Mariana Islands Department of Lands and Natural Resources Division of Fish and Wildlife. Common Food Fishes of Micronesia [Internet]. Available: http://www.cnmi-dfw.com/docs/Food_Fishes_Reef.pdf
